# Preclinical and clinical studies of estrogen deprivation support the PDGF/Abl pathway as a novel therapeutic target for overcoming endocrine resistance in breast cancer

**DOI:** 10.1186/bcr3191

**Published:** 2012-05-18

**Authors:** Marion T Weigel, Zara Ghazoui, Anita Dunbier, Sunil Pancholi, Mitch Dowsett, Lesley-Ann Martin

**Affiliations:** 1 Breakthrough Breast Cancer Centre, Institute of Cancer Research, 237 Fulham Road, London SW3 6JJ, UK; 2 Department of Academic Biochemistry, The Royal Marsden Hospital, Fulham Road, London, UK SW3 6JB, UK

## Abstract

**Introduction:**

The majority of breast tumors at primary diagnosis are estrogen receptor positive (ER+). Estrogen (E) mediates its effects by binding to the ER. Therapies targeting the estrogenic stimulation of tumor growth reduce mortality from ER+ breast cancer. However, resistance remains a major clinical problem.

**Methods:**

To identify molecular mechanisms associated with resistance to E-deprivation, we assessed the temporal changes in global gene expression during adaptation to long-term culture of MCF7 human breast cancer cells in the absence of estradiol (E2), long term estrogen deprived (LTED), that leads to recovery of proliferative status and models resistance to an aromatase inhibitor (AI). The expression levels of proteins were determined by western blotting. Proliferation assays were carried out using the dual platelet derived growth factor receptor (PDGFR)/Abelson tyrosine kinase (Abl) inhibitor nilotinib. Luciferase reporter assays were used to determine effects on ER-mediated transactivation. Changes in recruitment of cofactors to the gene regulated by estrogen in breast cancer 1 (GREB1) promoter were determined by chromatin immunoprecipitation (ChIP). Gene expression data were derived from 81 postmenopausal women with ER+ BC pre-treatment and at two-weeks post-treatment with single agent anastrozole in a neoadjuvant trial.

**Results:**

The PDGF/Abl canonical pathway was significantly elevated as early as one week post E-deprivation (*P *= 1.94 E-04) and this became the top adaptive pathway at the point of proliferative recovery (*P *= 1.15 E-07). Both PDGFRβ and Abl protein levels were elevated in the LTED cells compared to wild type (wt)-MCF7 cells. The PDGF/Abl tyrosine kinase inhibitor nilotinib, suppressed proliferation in LTED cells in the presence or absence of E. Nilotinib also suppressed ER-mediated transcription by destabilizing the ER and reducing recruitment of amplified in breast cancer-1 (AIB1) and the CREB binding protein (CBP) to the promoter of the E-responsive gene *GREB1*. High PDGFRβ in primary ER+ breast cancer of 81 patients prior to neoadjuvant treatment with an AI was associated with poorer antiproliferative response. Additionally PDGFRβ expression increased after two weeks of AI therapy (1.25 fold, *P *= 0.003).

**Conclusions:**

These preclinical and clinical data indicate that the PDGF/Abl signaling pathway merits clinical evaluation as a therapeutic target with endocrine therapy in ER+ breast cancer.

## Introduction

At primary diagnosis nearly 80% of breast cancers express estrogen receptor alpha (ERα) and proliferate in response to estrogen (E) [1]. Estrogen mediates its effects by binding to the ER, which subsequently associates with estrogen response elements (ERE) on target genes controlling proliferation and survival [2]. Classically, patients with ER+ breast cancer have been treated with endocrine agents, such as tamoxifen, which compete with E for the ER or aromatase inhibitors (AI), which block the conversion of androgens to estrogens [3]. Despite the efficacy of endocrine agents, both *de novo *and acquired resistance remain a significant clinical problem with up to 40% of patients relapsing on tamoxifen [4]. Although it was hoped that resistance to AIs would be less of a problem, many patients treated with AIs also exhibit resistance [4].

The molecular events that determine changes in the efficacy of endocrine therapies are not fully understood [5,6]. Preclinical and clinical studies provide support for mechanisms that involve cross-talk between ER and growth factor signaling pathways such as ERBB2/HER2 [5-7] but this is only overexpressed in about 10% of ER+ patients and is infrequently overexpressed with acquisition of resistance [8] indicating that alternative underlying molecular events remain to be discovered. *In vitro *models of resistance to endocrine therapy have relied on comparing the endocrine resistant cell lines with their isogenic wild type (wt) [9-16]. While this has provided valuable information highlighting many alterations in cell signaling, it has not addressed the temporal changes in genotype/phenotype that are directly associated with the acquisition of resistance. We used global gene expression analysis to assess the time-dependent changes in gene expression during the acquisition of resistance to estrogen deprivation using the ER+ breast cancer cell line MCF7. These data revealed the platelet derived growth factor (PDGF)/Ableson (Abl) canonical pathway as significantly upregulated as early as one-week post-estrogen deprivation and revealed this to be the top adaptive pathway at the point of full resistance. In studies of molecular changes occurring in tumors in a cohort of patients treated with an AI in the neoadjuvant setting we found PDGFRβ expression to be significantly associated with poor antiproliferative response to therapy. Finally nilotinib, a selective inhibitor of PDGF/Abl signaling was antiproliferative in LTED but not wt-MCF7 cells. These laboratory and clinical studies indicate that the PDGF/Abl signaling pathway is worthy of clinical targeting to reverse or restrict resistance to AIs.

## Materials and methods

### Cell culture and generation of the LTED cell line

The human ER-positive breast cancer cell line MCF7, obtained from American Type Culture Collection (Rockville, MD, USA), was cultured in phenol red-free Roswell Park Memorial Institute medium (RPMI) medium supplemented with 10% fetal bovine serum, 10 μg/ml insulin and 1nM estradiol (E2) and was referred to as wild-type MCF7. The wt-MCF7 cells were passaged weekly and medium was replenished every two to three days. To model acquisition of resistance to long term estrogen deprivation (LTED) on an AI, wt-MCF7 cells were cultured in phenol red-free RPMI medium supplemented with 10% dextran charcoal-stripped bovine serum (DCC) [17] and 10 μg/ml insulin. Monolayers were harvested at the time points indicated during acquisition of resistance. Over a period of 40 weeks LTED strains were generated in three independent experiments. For mechanistic studies LTED cells were passaged weekly and medium was replenished every two to three days. For all functional analysis cells were stripped of steroids and insulin over three days by culturing in phenol red-free RPMI 1640 supplemented with 10% DCC referred to as DCC-medium.

### Gene expression microarray analysis of cell lines

RNA was extracted from the LTED monolayers using RNeasy columns (Qiagen, Crawley, UK) according to the manufacturer's protocol. RNA amplification, labeling and hybridization on HumanWG-6 v3 Expression BeadChips (Illumina, San Diego, CA, USA) were performed according to the manufacturer's instructions [18] to assess changes in gene expression during adaptation to LTED. Nine time-points between one and 40 weeks were assessed. The HumanWG-6 v3 Expression BeadChip covers more than 48,000 transcript probes and its annotation is publicly available. Raw data were filtered and normalized using Robust Spline Normalization method (RSN) within the Lumi package in Bioconductor [19]. Probes were discarded from further analyses if they were not detected in any of the samples (detection *P*-value >1%). Differential gene expression analyses between the multiple time points were performed in a pairwise fashion using the BRB Array Tool [20]. Pathway analyses were performed on the differentially expressed genes using Ingenuity Pathway Analysis [21] (ArrayExpress Accession number, E-MTAB-922).

### Gene expression microarray analysis of breast tumors

Samples analyzed in this study were collected in the IL1839/223 study, which received approval from an institutional review board at each site and was conducted in accordance with the 1964 Declaration of Helsinki11 and International Conference on Harmonization/Good Clinical Practice guidelines. Written informed consent was obtained from each patient before participation including approval for the biological analysis of residual tissue samples [22]. Core-cut tumor biopsies were collected using 14-gauge needles from 81 postmenopausal women with stage I to IIIB ER+ early breast cancer before and after two weeks of anastrozole treatment in the anastrozole only arm of a neoadjuvant trial [22]. Total RNA was extracted using RNeasy (Qiagen, Crawley, UK). RNA quality was assessed using an Agilent Bioanalyzer (Santa Clara, CA, USA). Samples were analyzed only if their RNA integrity values were greater than 5.0. RNA amplification, labeling, and hybridization on HumanWG-6 v2 Expression BeadChips were performed according to the manufacturer's instructions (Illumina, San Diego, CA, USA). Raw data were filtered and normalized using the same method as performed for the cell lines.

A proliferation metagene consisting of 101 genes was developed by selecting the intersection of two proliferation clusters from two public breast cancer datasets [23,24]. These two clusters were derived from each of these two breast cancer datasets independently as the smallest clusters that contained 95% of the genes previously reported to be associated with proliferation [25-27]. Spearman correlation analyses were performed to assess the relationship between the baseline expression of PDGFRβ and change in the proliferation metagene.

### Proliferation assays

For proliferation assays, wt MCF7 cells were depleted of steroids for three days (stripped) by culturing in DCC-medium. Cells were subsequently seeded into 12-well plates at a density of 1 × 10^4 ^cells per well in DCC medium. LTED cells were treated similarly. Cells were left to acclimatize for 24 hours and were then treated with steroids or nilotinib (kindly provided by Novartis, Basel, Switzerland) for six days with daily changes. The cell number was determined using a Z1 Coulter counter (Beckman Coulter, High Wycombe, UK). Error bars represent ± standard error of the means (s.e.m.). Survival assays were compared using two-way analysis of variance (ANOVA) with Bonferroni correction.

### SiRNA knockdown studies

For siRNA knockdown, cells were plated in DCC medium on 96-well plates at a concentration of 2 × 10^3 ^(LTED) and 3 × 10^3 ^(MCF7) cells per well. After 24 hours cells were transfected with si control (non-targeting pool) or siRNA targeting *ABL, PDGFRB *or the combination, using DharmaFECT 3 (Dharmacon, Horsham, UK) according to the manufacturer's protocol. The following day transfected cells were treated with DCC medium alone or in the presence of E2 (0.01 nM). Cell growth was determined using CellTiter-glo luminescence assay (Promega, Southampton, UK) after 96 hours. Statistical analysis was performed using Student's T-test.

### Immmunoblotting

Cell monolayers were washed with ice-cold PBS and whole cell extracts generated as previously described [28]. Equal amounts of protein (50 μg) were resolved by SDS-PAGE and then subjected to immunoblot analysis. Antigen-antibody interactions were detected with ECL-reagent (Amersham, Amersham, UK). Phospho- and total-proteins were detected using the following antibodies: anti-ER (Novocastra, Milton Keynes, USA), anti-AKT, anti-phospho AKT, anti-phospho PDGFR β, anti-PDGFR β, anti-phospho Abl, anti-Abl (Cell Signaling Technologies, Danvers, MA, USA), anti-actin, anti-phospho ERK1/2 (Sigma, Gillingham, UK), anti-ERK1/2 (Santa Cruz, Santa Cruz, CA, USA).

### Transcription assays

Cell lines were stripped for three days and seeded in 24-well plates at a density of 7 × 10^4 ^cells per well for MCF7 and 5 × 10^4 ^cells per well for LTED in DCC medium. Twenty-four hours later monolayers were transfected using Fugene 6 (Roche, Burgess Hill, UK), with 0.1 μg of EREIItkluc (reporter) and 0.1 μg of pCH110 (β-galactosidase for normalization) according to the manufacturer's protocol, before treatment with the drugs indicated. After treatment for 24 hours the luciferase (Promega, Southampton, UK) and β-galactosidase (Galacto Star, Applied Biosystems, Bedford, MA, USA) activities were measured using a luminometer (TD20/20). Luciferase activity from triplicates was normalized and was expressed relative to vehicle treated control. Error bars represent ± s.e.m.

### QRT-PCR

Cells were plated at a density of 4 × 10^4 ^cells per well in 24-well plates into DCC medium. After 24 hours monolayers were transfected with siRNA as described above. RNA was extracted 48 hours later using the RNeasy kit (Qiagen). The mRNA was quantified using a NanoDrop 1000 spectrometer and reverse transcribed into cDNA using the SuperScript III First Strand Synthesis System for RT-PCR (Invitrogen, Grand Island, NY, USA). Expression levels of target genes were detected by qRT-PCR using Assay-on-Demand primer/probe sets *KIAA0674 *(Hs00391480-m1), *ABL1 *(Hs01104721-m1) and *PDGFRB *(Hs01019583-m1).

### ChIP analyses

LTED cells were treated with E2 alone or in combination with nilotinib for 45 minutes. Monolayers were fixed with 1% formaldehyde for ten minutes at room temperature and then quenched with glycine (0.125M). Chromatin was purified as previously described [28]. Chromatin complexes were immunoprecipitated with antibodies against ER (HC-20), AIB1 or CBP (Santa Cruz). Immune-complexes were purified and resulting DNA subjected to quantitative PCR analysis using SYBR green (Applied Biosystems, Bedford, MA, USA) in the presence of primers flanking the estrogen response element (ERE) within the promoter region of *GREB1 *(forward) 5'-GTGGCAACTGGGTCATTCTGA-3' and (reverse) 5'- CGACCCACAGAAATGAAAAGG-3'. Statistical analysis was performed using Student's T-test.

## Results

### PDGF/Abl canonical pathway is strongly associated with adaptation to long term estrogen deprivation (LTED)

**Figure 1:**
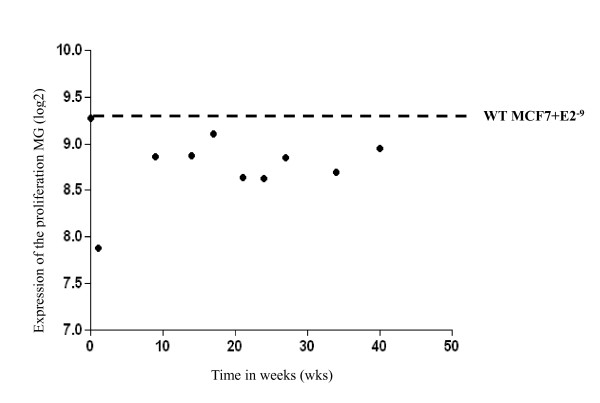
**Temporal changes in the expression of the proliferation metagene**. The dotted line represents the baseline proliferation metagene for the wt MCF7 cells cultured in E2 (1 nM). E2, estradiol; wt, wild type.

E-deprivation led to markedly decreased expression of the proliferation metagene (MG) after one week but near full recovery by nine weeks indicating resistance to E-deprivation by this time point (Figure 1). Thereafter, the expression of the MG remained stable. Moreover, global assessment of gene expression revealed stabilization of the gene signatures after this time-point. Based on this observation, further analyses were restricted to a triangular pairwise comparison of gene expression between wt MCF7 cells in the presence of 1 nM E2 (modeling pre-treatment), one week post E-deprivation (modeling early response to an AI) and at nine weeks post E-deprivation (modeling acquired resistance to an AI) (ArrayExpress Accession number, E-MTAB-922).

Comparison of gene expression in wt MCF7 cultured in the presence of E2 versus week one cells showed that 1,970 genes were down-regulated and 1,653 genes were up-regulated (*P *< 0.001/FDR = 0.004). The down-regulated genes were mainly associated with metabolic and cell cycle pathways (Table 1). The up-regulated genes were associated with interferon and JAK/STAT canonical pathways indicative of increased cell stress. Comparison of week one versus week nine revealed 239 down-regulated genes and 236 up-regulated genes (*P *< 0.001/FDR = 0.03). Many of the down-regulated genes were involved in immune response pathways. The major up-regulated canonical pathways were involved in metabolism and cell cycle apparently reversing many of the changes seen by week one and consistent with the increase of the expression of the proliferation metagene.

**Table 1:** Comparison of the canonical pathways

UP-REGULATED	*P *value	DOWN-REGULATED	*P *value
*Comparison A: Wt MCF7+E2 versus week 1*		*Comparison A: Wt MCF7+E2 versus week 1*	

Interferon signalling	2.79 E-07	Purine metabolism	**1.13 E-12**
Molecular mechanisms of cancer	1.17 E-05	Pyrimidine metabolism	**2.11 E-10**
JAK/STAT signalling	2.72 E-05	Protein ubiquitination pathway	**6.48 E-08**
Germ cell-steroli cell junction signalling	2.76 E-05	Role of BRCA1 in DNA damage response	**6.92 E-08**
Inositol phosphate metabolism	1.15 E-04	Mitotic roles of polo-like kinase	**5.72 E-07**
IL-3 signalling	1.63 E-04	Role of CHK proteins in cell cycle checkpoint control	**1.41 E-06**
PDGF signalling	1.94 E-04	ATM signalling	**3.06 E-06**
Neuregulin signalling	2.15 E-04	Cell cycle:G2/M DNA damage checkpoint regulation	**1.23 E-05**
Erythropoietin signalling	2.31 E-04	Cleavage and polyadenylation of pre-mRNA	**1.24 E-04**
PI3K/AKT signalling	2.95 E-05	Alanine and aspartate metabolism	**5.15 E-04**

*Comparison B: Week 1 versus week 9*		*Comparison B: Week 1 versus week 9*	

Mitotic roles of polo-like kinase	4.71E-08	Antigen presentation pathway	**8.88E-20**
Role of CHK proteins in cell cycle checkpoint control	2.08E-07	Interferon signalling	**1.44E-17**
ATM signalling	3.27E-07	Activation of IRF by cytosolic pattern recognition receptors	**2.48E-10**
Role of BRCA1 in DNA damage and response	8.75E-06	Role of pattern recognition receptors in recognition of bacteria and viruses	**2.37E-07**
Hereditary breast cancer signalling	2.08E-05	Dendritic cell maturation	**3.54E-06**
Pyrimidine metabolism	3.34E-05	Allograft rejection signalling	**7.64E-06**
Cell cycle: G2/M DNA damage checkpoint regulation	1.57E-04	Autoimmune thyroid disease signalling	**1.04E-05**
		Graft-versus-host disease signalling	**1.21E-05**
		Role of RIG1-like receptors in antiviral innate immunity	**1.39E-05**
		Cross talk between dendritic cells and natural killer cells	**2.02E-05**

*Comparison C: Wt MCF7+E2 versus week 9*		*Comparison C: Wt MCF7+E2 versus week 9*	

PDGF signalling	1.15E-07	Protein ubiquitination pathway	**1.63 E-07**
Molecular mechanisms of cancer	1.53E-07	Purine metabolism	**6.49 E-07**
Actin cytoskeleton signalling	4.92E-07	Pyrimidine metabolism	**7.92 E-06**
Integrin signalling	6.93E-07	Mitochondrial dysfunction	**1.25 E-04**
PI3K/AKT signalling	1.06E-06	Oxidative phosphorylation	**2.47 E-04**
EGF signalling	5.88E-06	Citrate cycle	**4.79 E-04**
FAK signalling	8.45E-06	Role of BRCA1 in DNA damage response	**7.53 E-04**
HGF signalling	1.23E-05	Lysine degradation	**7.84 E-04**
14-3-3 mediated signalling	1.30E-05		
Neuregulin signalling	1.53E-05		
Ephrin Receptor Signaling	1.66E-05		
IGF-1 Signaling	1.85E-05		
ILK Signaling	2.27E-05		
Axonal Guidance Signaling	3.12E-05		
JAK/Stat Signaling	6.14E-05		
Insulin Receptor Signaling	6.17E-05		

ATM, ataxia teleangiectasia mutated; BRCA1, breast cancer type 1 susceptibility protein; CHK, csk-homologous kinase; E2, estradiol; EGF, epidermal growth factor; FAK, focal adhesion kinase; HGF, hepatocyte growth factor; IGF-1, insulin-like growth factor 1; IL-3, interleukin 3; ILK, integrin-linked kinase; IRF, interferon regulatory factor; JAK, Janus kinase; PDGF, platelet-derived growth factor; PI3K, phosphatidylinositol 3-kinase; RIG1, retinoid-inducible gene 1; STAT, Signal Transducer and Activator of Transcription;

Given the relatively similar proliferation status of wt MCF7 and week nine cells it was rationalized that comparison of wt MCF7 versus nine weeks E-deprivation would negate the overriding effect of the proliferation signature and unmask the underlying adaptive changes associated with acquired resistance. This analysis revealed 1,753 down-regulated genes and 1,758 up-regulated genes (*P *< 0.001/FDR = 0.0048). All major up-regulated canonical pathways were involved in classical cell signaling including PI3K/AKT/p70S6 and IGF1. Of particular interest the PDGF/Abl canonical pathway was significantly elevated as early as one-week post E-deprivation (*P *= 1.94 × 10^-4^) and was the top adaptive pathway at the point of resistance (*P *= 1.15 × 10^-7^). In order to assess the relevance of the PDGF/Abl pathway we interrogated global gene transcription data from two publically available global gene transcription data sets from ER+ breast cancer cell lines (ZR-75.1, MDA MB 361 and HCC-1428) that had been adapted to LTED [11,15]. The PDGF/Abl pathway was not significantly altered within these data sets. Of note, unlike our LTED cells many of these cell lines lost or significantly reduced expression of ER during adaptation to estrogen deprivation. In order to determine the validity of our findings we, therefore, generated a further set of MCF7-LTED cells and found that the PDGF/Abl pathway was once again significantly increased indicating that acquisition of this pathway may be context specific.

### PDGFR expression is significantly associated with poor anti-proliferative response to aromatase inhibition in primary ER+ breast carcinomas

**Figure 2:**
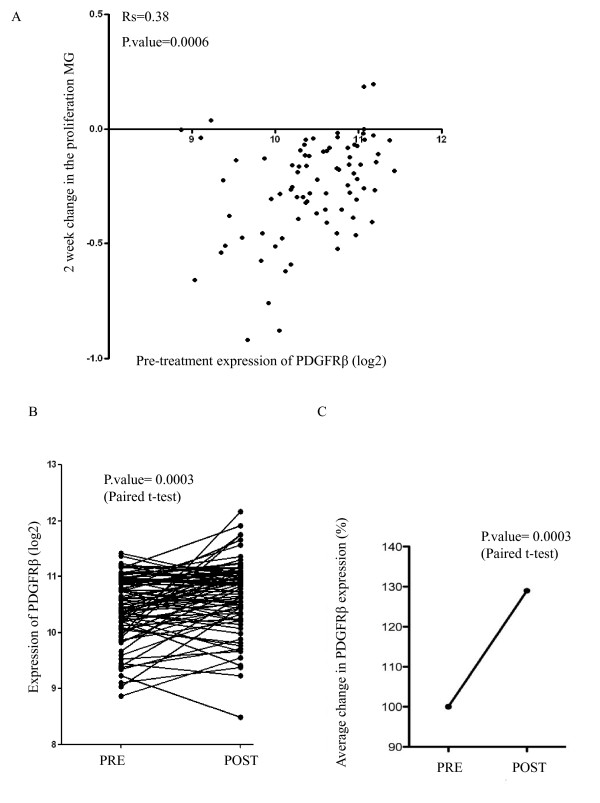
**Expression of PDGFRβ in clinical samples**. **A**. Baseline expression of PDGFRβ versus two-week change in the proliferation metagene in 81 breast cancer patients. **B**. Expression changes of PDGFRβ in response to AI treatment. C. Average percentage change in PDGFRβ expression pre- and post-AI treatment. AI, aromatase inhibitor; PDGFRβ, platelet derived growth factor receptor β.

Prior to further molecular characterization of the PDGF/Abl pathway as a putative mechanism of resistance and potential target for therapy we sought evidence for its likely clinical relevance by interrogating global gene expression data from 81 ER+ patients who received the AI anastrozole as neoadjuvant (pre-surgical) treatment for 14 weeks. After two weeks on treatment, a second biopsy was taken to generate paired gene expression profiles during estrogen deprivation. *PDGFRB *showed no significant correlation with known predictive markers of hormone sensitivity such as ER or progesterone receptor (PGR) at baseline. No significant correlation with Ki67 or tumor size was observed. However, *PDGFRB *associated negatively with the Global Index of Dependence on Estrogen (GIDE) (r = 0.41 and *P *= 0.0002) [29] that reflects the overall transcriptional response to E-deprivation. Furthermore, high *PDGFRB *levels pre-treatment were significantly associated with a poor response to the AI as measured using the proliferation metagene (*P *= 0.0006) (Figure 2A).

Additionally, pairwise comparison showed a significant increase in *PDGFRB *and *PDGFRL *expression after two weeks of estrogen deprivation (1.25-fold, *P *= 0.0003, 1.43-fold, *P *< 0.001), respectively (Figure 2B and 2C). The increase in *PDGFRB *and *PDGFRL *in response to neoadjuvant AI was further confirmed in an external clinical dataset from patients treated with two weeks of neoadjuvant letrozole (1.2-fold, *P *= 0.009 and 1.51-fold, *P *< 0.001) [30].

### The PDGF/Abl pathway acts as a driver of proliferation in the LTED cells

**Figure 3:**
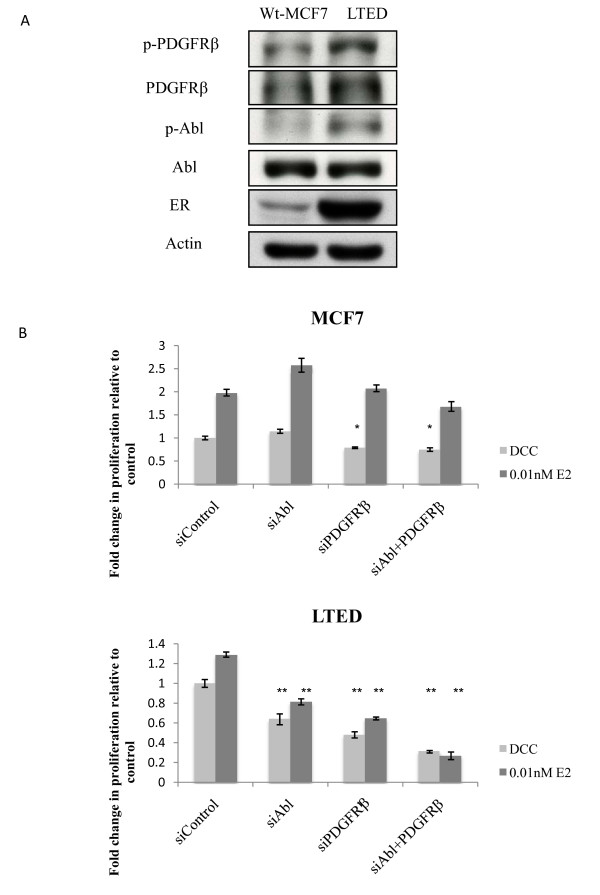
**Expression of PDGFRβ and Abl in breast cancer cell lines**. **A**. Wt MCF7 and LTED cells were screened for kinase expression using western blotting. Cells were grown under basal conditions. Whole cell extracts were probed using antibodies against the indicated proteins. **B**. Wt MCF7 and **C**. LTED cells were seeded into 96 well plates and transfected with small interfering RNA (siRNA) control, siPDGFRβ, siAbl or the combination of both and then treated ± E2 for 96 hours. Proliferation was measured using Cell Titre glo. Data are representative of three individual experiments. Error bars express ± SEM * *P *< 0.05, ** *P *< 0.01. Abl, Abelson tyrosine kinase; E2, estradiol; LTED, long term estrogen deprived; PDGFRβ, platelet derived growth factor receptor β; SEM, standard error of the mean; siRNA small interfering RNA; Wt, wild type.

Given the striking observation that PDGF/Abl was identified as the top adaptive pathway, expression levels of proteins included in the PDGF/Abl signaling pathway were investigated in the model systems. When compared to the wt-MCF7, the LTED cells expressed higher levels of PDGFRβ Moreover, an up-regulation of both PDGFRβ and Abl phosphorylation was detected in the LTED cell line. PDGFRα expression could not be detected (Figure 3A).

To determine the relevance of single or combined PDGFR and Abl inhibition on cell proliferation, short-term (96 hours) siRNA knockdowns for the individual proteins or their combination were performed in MCF7 and LTED cells. The efficiency of the siRNA knockdown was assessed by qRT-PCR [See Additional file 1, Figure S1]. In the MCF7 cells neither PDGFRβ nor Abl knockdown had a significant inhibitory effect on proliferation in the presence or absence of E2. In contrast, knockdown of Abl in LTED cells reduced proliferation significantly in both the absence and presence of E2. An even greater anti-proliferative effect was observed using a siRNA knockdown of PDGFRβ in LTED cells in the absence and presence of E2. Of note, the combined knock down of both targets suppressed proliferation even further providing additional evidence of the relevance of this canonical pathway in adaptation to LTED (Figure 3B and 3C).

**Figure 4:**
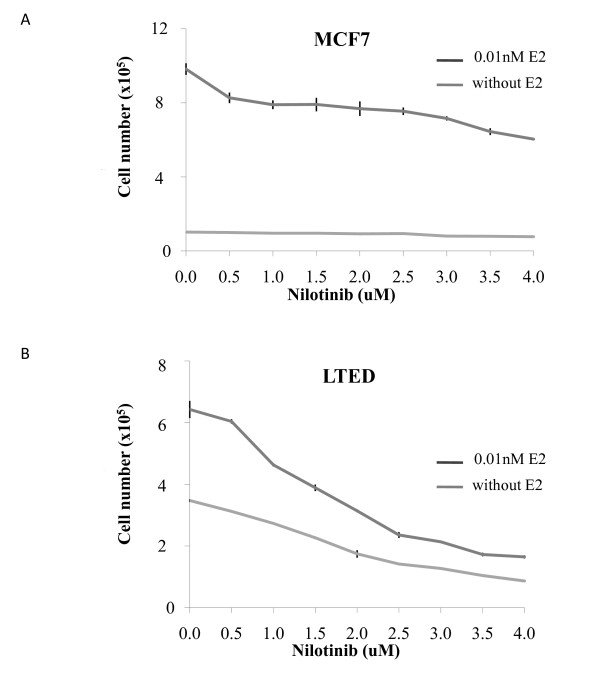
**Inhibition of PDGFRβ and Abl using nilotinib suppresses growth of LTED cells**. The endocrine sensitive cell line MCF7 (**A**) and the resistant cell line LTED (**B**) were seeded on 12-well plates and incubated for six days with various concentrations of nilotinib ± E2. Cell proliferation was determined using a Coulter counter. Data are representative of three individual experiments. Error bars express ± SEM of triplicate samples. Two way ANOVA showed a significant difference in cell survival in the LTED cells in the presence or absence of E2 at doses of 1 uM and above *P *< 0.0001. Abl, Abelson tyrosine kinase; ANOVA, analysis of variance; E2, estradiol; LTED, long term estrogen deprived; PDGFRβ, platelet derived growth factor receptor β; SEM, standard error of the mean.

To establish the therapeutic relevance of the PDGF/Abl pathway, we assessed the effect of nilotinib, a dual PDGFR and Abl inhibitor on cell proliferation. Increasing concentrations of nilotinib caused a slight but noticeable decrease in proliferation (Figure 4A and 4B) in wt MCF7 cells in the presence of 0.01 nM estradiol (E2). However, this did not meet an IC50. Assessment of the anti-proliferative effect of nilotinib in the absence of E2 provided no further benefit compared to E2-deprivation alone. In contrast in the LTED model, nilotinib inhibited cell proliferation significantly both in the presence and absence of E2. The IC_50 _dose of nilotinib in this model (2 μM) is within the specific dose range achievable *in vivo *[31].

### Inhibition of PDGFR/Abl reduces ER protein levels

**Figure 5:**
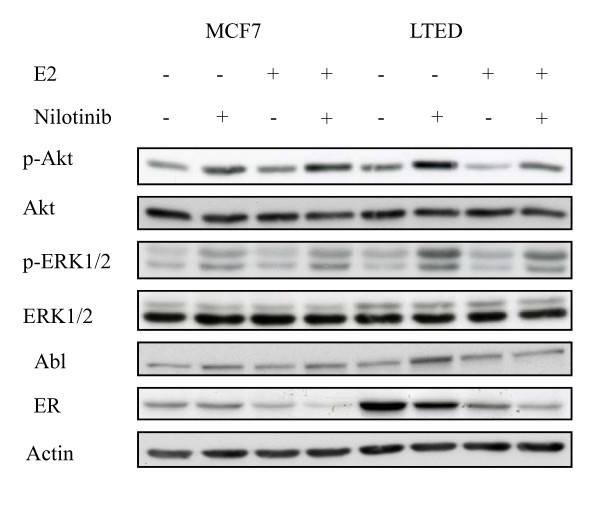
**Inhibition of PDGFRβ and Abl with nilotinib reduces ER protein stability**. Wt MCF 7 and LTED cells were treated with nilotinib (2 µM) ± E2 for 24 hours. Cell monolayers were subsequently harvested and whole cell extracts probed for the proteins indicated. Data shown are representative of three independent experiments. Abl, Abelson tyrosine kinase; E2, estradiol; LTED, long term estrogen deprived; Wt, wild type.

To elucidate the mechanism by which suppression of PDGFR/Abl mediated the anti-proliferative effect seen, wt MCF7 and LTED cells were treated with nilotinib in the presence and absence of E2 and effects on downstream signaling were assessed. In the presence of nilotinib, phosphorylation of both AKT and ERK1/2 was elevated irrespective of the presence of E2. Similarly, nilotinib induced a slight but noticeable increase in Abl. Strikingly, treatment with nilotinib suppressed the level of ER in both cell lines although this was more noticeable in the LTED cells (Figure 5).

### Inhibition of PDGFR/Abl signaling suppresses ER-mediated transcription in LTED cells

**Figure 6:**
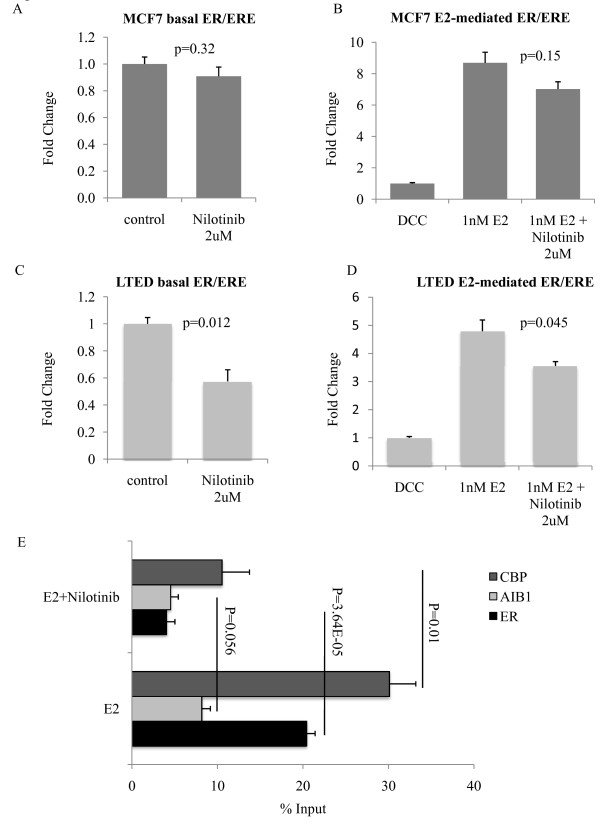
**Inhibition of PDGFR/Abl suppresses ER/ERE-mediated transactivation**. Cells were transfected with an artificial luciferase reporter and treated as indicated. Luciferase activity was measured and normalized to DCC to quantify ER-transactivation. **A **and **B **show the effect of nilotinib on ER-mediated transcription in wt-MCF7. **C **and **D **show the effect of nilotinib on ER-mediated transcription in LTED cells. The data shown are representative of four individual experiments. Bars represent ± SEM of triplicate samples. **E**. ChIP analysis to determine ER, AIB1 and CBP recruitment to the *GREB1 *promoter was performed. Cell monolayers were serum starved for 24 hours and treated for 45 minutes with E2 (1 nM) and nilotinib (4 μM) as indicated. Antibodies against total ERα, AIB1 and CBP were used to pull-down protein complexes and to assess their recruitment to the ERE located in the *GREB1 *promoter by q-PCR. Data shown are representative of two independent experiments. Abl, Abelson tyrosine kinase; AIB1, amplified in breast cancer 1; CBP, CREB binding protein; ChIP, chromatin immunoprecipitation; DCC, dextran charcoal-stripped bovine serum; E2, estradiol; ER, estrogen receptor; ERE, estrogen response element; LTED, long term estrogen deprived; SEM, standard error of the mean; wt, wild type.

To elucidate the effect of PDGFR/Abl inhibition on ER-mediated transcription, cells were transiently transfected with the ERE-luciferase reporter construct and treated with nilotinib in the presence and absence of E2 (Figure 6). In the wt-MCF7 cells nilotinib had no effect on ER/ERE-mediated transactivation in the absence of E2 and a slight, but minimal inhibitory effect in the presence of E2 (Figure 6 A, B). However, in the LTED cells ER/ERE-mediated transactivation was significantly inhibited in the absence of E2. Addition of E2 reduced the inhibitory effect of nilotinib on ER-mediated transcription, data in parallel with the effects on proliferation (Figure 6 C, D).

We next examined the effect of nilotinib on the recruitment of ER together with its co-activators to the promoter of *GREB1 *(an endogenous E-regulated gene) using chromatin immunoprecipitation (ChIP). As ER/ERE-mediated transcription was unaffected by nilotinib in the wt-MCF7 we focused our attention on the LTED cells. Nilotinib in the presence of E2 decreased the recruitment of ER, AIB1 and the CREB binding protein (CBP) to the ERE located within the *GREB1 *promoter compared to E2 alone (Figure 6E).

## Discussion

Resistance to endocrine therapy is a major clinical problem in the treatment of breast cancer. Previously we, along with others, have highlighted the role of cross-talk between the ER and ERBB2/HER2 signaling pathways leading to endocrine resistance as a result of ligand-independent activation of the ER or by the generation of an E-hypersensitive phenotype [10,14,32-35]. In the current study, we used a novel strategy to try to identify temporal changes in the transcriptome associated with the acquisition of resistance to LTED. Using a proliferation MG we showed that proliferation recovered, that is, resistance occurred, as early as nine weeks post E-deprivation.

Comparison of the wt-MCF7 cells (modeling pre-treatment) versus MCF7 cells after one week of E-deprivation (modeling a patient responding to an AI) highlighted proliferation and metabolic canonical pathways as the most significantly down-regulated. This is in keeping with previous short term clinical investigations of AIs [29,36,37]. Of note, the JAK/STAT and interferon canonical pathways were markedly up-regulated after one week of E-deprivation. It has been shown that signal transducer and activator of transcription 1 (STAT1) is particularly important in activating IFN-γ and its antitumor effects. In addition to inhibiting proliferation and survival, IFN-γ enhances the immunogenicity of tumor cells, in part, by enhancing the STAT1-dependent expression of MHC proteins [38].

Changes in transcription profiles after one week of E-deprivation with nine weeks of E-deprivation (modeling a patient with acquired endocrine resistant disease) in part reflected the reinstatement of proliferation, but also showed that IFN signaling and several canonical pathways associated with immune recognition were down regulated over that period. Clinical studies have shown that in triple negative breast cancer impaired immune response might be linked with the development of distant metastases. Indeed, high expression of an immune response gene expression module was associated with a significantly better outcome in two independent studies [39,40]. Our data suggest that part of these immune signatures may emanate from epithelial cells and not from an inflammatory infiltrate.

Comparison of the wt MCF7 cells with nine weeks post E-deprivation negated the overriding effect of the proliferation signature and unmasked the underlying adaptive changes associated with acquired resistance. The major up-regulated canonical pathways were identified as all being classically associated with cell signaling including PI3K/AKT/p70S6 and IGF1 all of which have been associated with poor prognosis in previous studies of ER+ breast cancer [41-46]. Of particular note, two recent clinical studies TAMRAD [47] and BOLERO-2 [48] have reported substantially greater activity of the mTOR inhibitor everolimus (RAD001) in the metastatic setting after relapse on AI therapy.

Our most striking observation, however, was the alteration in PDGF/Abl signaling. This canonical pathway was elevated as early as one week post E-deprivation. Although the over riding effect of E-deprivation after one week was suppression of proliferation, pathways such as PDGF/Abl, neuregulin (Her2) and PI3 kinase were up-regulated and may be indicative of early adaptive responses pre-dating cell growth. Surprisingly, PDGF/Abl became the top adaptive pathway at the point of resistance superseding both the neuregulin and PI3 kinase canonical pathways, both of which have been previously reported to be strongly associated with endocrine resistance [15].

PDGF receptors (PDGFR) contain an intracellular tyrosine kinase domain whose activation is dependent on binding of PDGF resulting in stimulation of several intracellular pathways, leading to cell proliferation and survival [49]. PDGF can promote tumor growth via autocrine stimulation of malignant cells, overexpression or overactivation of PDGFRs, or by stimulating tumor angiogenesis. For this reason, targeting PDGF signaling has become of interest for the development of anticancer therapeutics. Two main approaches have been taken to inhibit PDGFR signaling in cancer: direct targeting of tumor cells proliferating in response to PDGF signaling or indirect inhibition of tumor growth by targeting pericytes to decrease angiogenesis [49]. Abl is a Src-like nonreceptor protein kinase that acts down-stream of the PDGFR. Abl is involved in the regulation of cell proliferation, apoptosis, adhesion, cell migration and stress response [50,51]. The existence of C-terminal DNA-binding motifs and nuclear localization signals enables Abl to shuttle between cytoplasmic and nuclear compartments [52,53]. Activating translocations of *ABL*, such as *BCR-ABL*, are pivotal for the development of chronic myelogenous leukemia [54].

To determine the clinical significance of our finding we used global gene transcription data from a cohort of patients with ER+ primary breast cancer before and after two weeks of neoadjuvant AI therapy. Notably *PDGFRB *and *PDGFRL *expression was increased after two weeks of E-deprivation (Figure 2B). Moreover, low *PDGFRB *levels pre-treatment were associated with a better response to the AI. This would support the possibility that expression of PDGFRβ may be an early marker of *de novo *and/or acquired endocrine resistance. In support of our finding a recent clinical study showed that elevated levels of stromal PDGFRβ were associated with a poor prognosis in breast cancer patients [55].

To investigate the role of PDGF/Abl signaling during adaptation to LTED we selected the dual PDGFR/Abl kinase inhibitor nilotinib. Treatment of the wt MCF7 cells in the presence of E2 modeling a patient at primary diagnosis showed that nilotinib as a monotherapy caused a concentration-dependent decrease in proliferation but this was far less compared with E-deprivation alone. In order to model the effect of nilotinib in patients who have relapsed on an AI and for whom treatment has ceased we treated the LTED cells with 0.01 nM E2. In keeping with our previous data [56] we showed that E2 caused an increase in proliferation (1.85-fold) although the magnitude of the response was far less than that seen in the MCF7 cells (12-fold). The LTED cells were significantly sensitive to nilotinib in the presence of E2. The IC_50 _dose of nilotinib in this model was within the range of plasma levels achieved clinically. Most strikingly, proliferation was substantially lower with nilotinib in the absence of E2. This suggests that in the clinical setting nilotinib may be useful in combination with an AI to delay the onset of resistance or indeed to prolong the efficacy of the AI in the metastatic setting.

In order to determine whether PDGFRβ or Abl was dominant in the LTED phenotype, we used siRNA knockdown. Surprisingly both kinases appeared integral to the phenotype, although suppression of PDGFRβ inhibited proliferation to a greater degree. Assessment of the effect of nilotinib on downstream signaling in both the wt MCF7 and the LTED in the presence and absence of E2 showed that nilotinib increased both AKT and ERK1/2 phosphorylation. Studies with dasatinib, which targets Src-family kinases and Abl have shown similar increases in signaling via these pathways. It has been postulated that this might be indicative of an early resistance mechanism to inhibition of these non-receptor tyrosine kinase pathways [57].

Of particular note, nilotinib significantly decreased ER levels. It has been demonstrated using transient transfection that Abl regulates ER protein stability via phosphorylation of tyrosine 52 and 219 [58]. Similarly, Abl has been shown to phosphorylate AIB1, a nuclear co-activator for ER, providing further evidence for the role of Abl in modulating ER genomic function [59]. As the LTED cells remain dependent on the ER for proliferation [12] we hypothesized that inhibition of Abl may suppress ER-mediated transcription. Indeed, we were able to demonstrate that nilotinib significantly reduced ER/ERE transactivation as a result of decreased recruitment of ER, AIB1 and CBP (Figure 6).

These data suggest that PDGFR/Abl signaling may provide a therapeutic target in ER+ breast cancer. Recently, the clinical significance of impeding c-kit and PDGFR in combination with aromatase inhibition has been addressed in two single arm clinical trials in ER+ breast cancer patients [60,61]. In the first pre-operative study ER+ patients were treated with letrozole plus imatinib, a c-Kit/PDGFR/Abl receptor tyrosine kinase inhibitor, for three months. Of the ten evaluable patients, nine achieved clinical partial response and one had stable disease. In a second single arm study, postmenopausal women with ER+ disease and no prior endocrine therapy for metastatic breast cancer who expressed PDGFR and/or c-kit, were treated with letrozole plus imatinib. Partial response was achieved in seven patients (15.6%) and stable disease was observed in 20 patients (44%). The disadvantage of these studies is that the AI alone was not assessed and, therefore, it is impossible to ascertain the benefit gained by the combination. To address this, a two arm study comparing an AI versus AI plus imatinib or nilotinib would be required in which patients with ER+/PDGFR+ breast cancer would be eligible.

## Conclusions

Using temporal global gene expression data together with functional analysis we have identified a novel interaction between ER and the PDGF/Abl signal transduction pathway that occurs during adaptation to LTED and which appears partly responsible for the resistant phenotype. One of the major limitations of this study is the use of a single cell line model of acquired resistance to E-deprivation and as such these finding may be context specific. However, we were able to confirm the clinical relevance of these *in vitro *observations in two independent and heterogeneous cohorts of patients treated with an AI.

Taken together these data suggest that PDGFRβ may provide a novel biomarker of early resistance to AI therapy and with further *in vitro *and *in vivo *validation may prove to be a novel therapeutic target in treatment and/or avoidance of endocrine resistance in certain patient populations.

## Abbreviations

Abl: Abelson tyrosine kinase; AI: aromatase inhibitor; AIB1: amplified in breast cancer 1; CBP: CREB binding protein; ChIP: chromatin immunoprecipitation; DCC: dextran charcoal-stripped bovine serum; E2: estradiol; ERBB2: human epidermal growth factor receptor 2; ER: estrogen receptor; ERE: estrogen response element; GREB1: gene regulated by estrogen in breast cancer 1; IFN: interferon; IGF1: insulin like growth factor; LTED: long term estrogen deprived; MG: metagene; PBS: phosphate buffered saline; PDGFR: platelet derived growth factor receptor; PI3K: phosphatidylinositol 3-kinases; RT-PCR: reverse transcriptase polymerase chain reaction; s.e.m.: standard error of the mean; siRNA: small interfering RNA; STAT: signal transducer and activator of transcription; wt: wild type.

## Competing interests

MTW, ZG, AD and SP have no conflict of interest that could be perceived as prejudicing the impartiality of the research reported; MD receives research funding, honoraria for advisory boards and lecture fees from AstraZeneca, Novartis and Roche: LAM receives academic research funding from AstraZeneca and Pfizer.

## Authors' contributions

MTW was involved in the design of the study, carried out the *in vitro *research and helped draft the manuscript. ZG carried out the bioinformatics and helped draft the manuscript. AD was involved in the bioinformatics analyses. SP helped generate the LTED cell line and was involved in the molecular research. MD critically reviewed the manuscript. LAM developed the concept and experimental design, generated the LTED cell line, carried out the ChIP assays and wrote the manuscript. All authors have read and approved the final manuscript.

## Supplementary Material

Additional file 1 **Figure S1: siRNA knockdown of PDGFRβ and Abl reduces mRNA expression**. Cells were transfected with siRNA against PDGFRβ, Abl or the combination of the two. Forty-eight hours after transfection mRNA was extracted, quantified and reverse transcribed. Expression levels of genes were detected using qRT-PCR. Error bars represent ± SEM. Click here for file
