# Supplementary figures and images for: Preclinical and clinical studies of estrogen deprivation support the PDGF/Abl pathway as a novel therapeutic target for overcoming endocrine resistance in breast cancer
Source: Breast Cancer Res. 2012 May 18;14(3):R78. doi: 10.1186/bcr3191 (PMC3446341; doi:10.1186/bcr3191)

## Slide 1
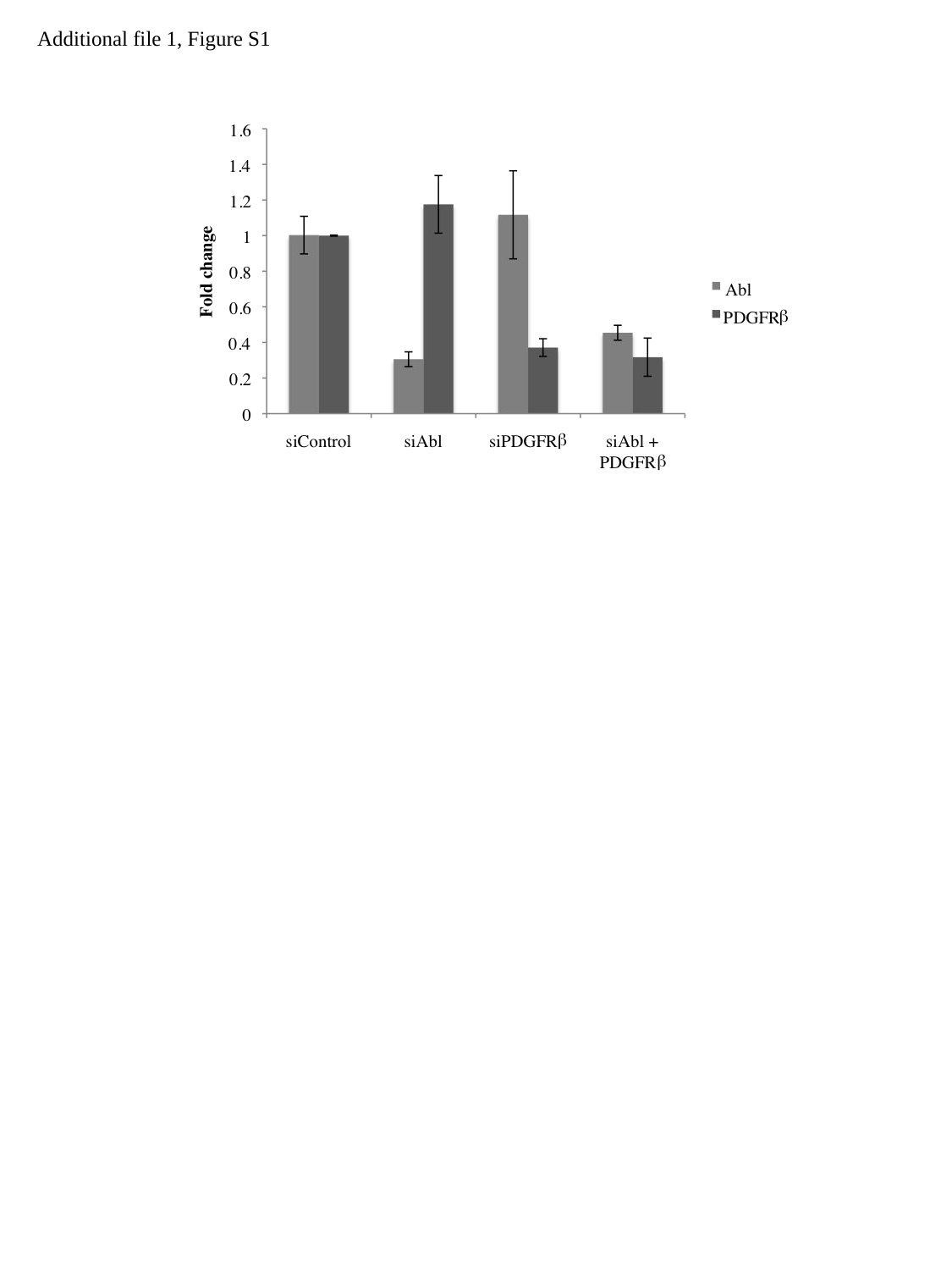

Additional file 1, Figure S1
β
β
β

Supplement: Additional file 1 — Figure S1: siRNA knockdown of PDGFRβ and Abl reduces mRNA expression. Cells were transfected with siRNA against PDGFRβ, Abl or the combination of the two. Forty-eight hours after transfection mRNA was extracted, quantified and reverse transcribed. Expression levels of genes were detected using qRT-PCR. Error bars represent ± SEM. [file bcr3191-S1.PPT]
